# Updated overall survival in patients with prior checkpoint inhibitor therapy in the phase III TIVO-3 study

**DOI:** 10.1093/oncolo/oyae369

**Published:** 2025-02-06

**Authors:** Miguel Zugman, David F McDermott, Bernard J Escudier, Thomas E Hutson, Camillo Porta, Elena Verzoni, Michael B Atkins, Brian Rini, Sumanta K Pal

**Affiliations:** Department of Medical Oncology & Experimental Therapeutics, City of Hope Comprehensive Cancer Center, Duarte, CA, 91010, United States; Division of Medical Oncology, Beth Israel Deaconess Medical Center, Dana-Farber/Harvard Cancer Center, Boston, MA, 02215, United States; Department of Medical Oncology, Gustave Roussy, Villejuif, 94 805, France; Hematology and Medical Oncology, UMC Cancer Center, Texas Tech University Health Science Center School of Medicine, Lubbock, Texas, 79430, United States; Interdisciplinary Department of Medicine, University of Bari Aldo Moro and Division of Medical Oncology, A.O.U. Consorziale Policlinico di Bari, Bari, 70124, Italy; Genitourinary Medical Oncology, Fondazione IRCCS Istituto Nazionale Tumori, Milan, 20133, Italy; Department of Oncology, Georgetown Lombardi Comprehensive Cancer Center, Washington, DC, 20007, United States; Department of Medicine, Vanderbilt-Ingram Cancer Center, Nashville, TN, 37232, United States; Department of Medical Oncology & Experimental Therapeutics, City of Hope Comprehensive Cancer Center, Duarte, CA, 91010, United States

**Keywords:** metastatic renal cell carcinoma, tivozanib, sorafenib, vascular endothelial growth factor inhibitor, immunotherapy

## Abstract

**Background:**

The phase III TIVO-3 study demonstrated improvement in progression-free survival (PFS) with tivozanib compared with sorafenib in patients with 2-3 prior systemic regimens for metastatic renal cell carcinoma (mRCC).

**Methods:**

The TIVO-3 trial enrolled patients with measurable mRCC who had received 2 or more prior systemic therapies, including a vascular endothelial growth factor tyrosine kinase inhibitor (VEGF-TKI). Patients were stratified by International Metastatic RCC Database Consortium risk score and type of prior treatment and were randomized 1:1 to receive tivozanib or sorafenib. Efficacy was assessed using Response Evaluation Criteria in Solid Tumors version 1.1 criteria, with PFS as the primary endpoint. Safety was evaluated using Common Terminology Criteria for Adverse Events version v4.03, and statistical analyses included Cox regression for overall survival (OS) and descriptive statistics for duration of response (DOR). The current post-hoc long-term follow-up analysis consists of an assessment of OS in the previously stratified subpopulation of patients with prior CPI exposure.

**Results:**

Between May 2016, and August 2017, 350 patients were randomized, of which 26% had prior CPI exposure, with final analysis data cut off on June 21, 2021. In patients previously treated with CPIs (*n* = 91), the median PFS of tivozanib was 7.3 months versus 5.1 months with sorafenib and hazard ratio (HR) of 0.55 (95% CI, 0.32-0.94). The OS HR in the CPI-treated subset was 0.69 (95% CI, 0.43-1.11, *P* =.0992) favoring tivozanib, although with a median OS of 18.1 and 20.9 months, for tivozanib and sorafenib, respectively. Tivozanib demonstrated a longer median DOR of 20.3 versus 5.7 months for sorafenib in the subset previously treated with CPIs. The safety profile favored tivozanib, with lower rates of VEGF-TKI class-related grade ≥3 adverse events compared with sorafenib. However, in the subset of patients previously treated with CPIs, the incidence of grade ≥3 adverse events was higher, at 58% for tivozanib and 67% for sorafenib, compared with the ITT population, at 46% and 55%, respectively.

**Conclusions:**

In this long-term post-hoc update of the TIVO-3 trial, we show that in CPI-resistant mRCC, the PFS benefit of tivozanib over sorafenib is accompanied with improved OS data, although not statistically significant, and durable responses.

**Implications for Practice**

TIVO-3 is a phase III study comparing tivozanib to sorafenib in patients previously treated with 2 or more systemic therapies, including at least 1 VEGF-directed therapy. We have previously reported that the study met its primary endpoint, showing a benefit in progression-free survival (PFS) with tivozanib over sorafenib. In this updated analysis, we focus on the subset of patients who received prior checkpoint inhibitors, reflecting a more contemporary group of patients. In this subset, a benefit in PFS was observed as well as a nonsignificant trend toward benefit in overall survival, reinforcing tivozanib as a viable option in this setting.

## Introduction

The treatment landscape of metastatic renal cell carcinoma (mRCC) has considerably evolved over the last decade. With the advent of immune checkpoint inhibitors (CPIs), several combinations have demonstrated superior outcomes in clinical trials. These include tyrosine kinase inhibitors (TKIs) targeting vascular endothelial growth factor (VEGF) used with CPIs (eg, axitinib with pembrolizumab, cabozantinib with nivolumab, or lenvatinib with pembrolizumab), as well as dual CPI combinations (eg, nivolumab with ipilimumab).^1-4^ Despite the improved efficacy of these regimens, many patients still present with disease that does not respond or eventually progresses after these treatments. While evidence supports the sequential use of VEGF-TKIs after disease progression following prior VEGF-TKI treatment, less data are available for treating CPI-resistant disease.^5-7^

Recently, data have been published for belzutifan, a hypoxia inducible factor-2 alpha (HIF-2 alpha) inhibitor, in the second or later line of therapy after disease progression following both VEGF-TKIs and CPIs, in comparison to everolimus, a mammalian target of rapamycin (mTOR) inhibitor.^8^ Before that, the TIVO-3 (NCT02627963) study had been the only published phase III trial of a VEGFR-TKI with positive results addressing treatment for patients who have progressed on 2 or more systemic therapies, including VEGF-TKIs, and also CPIs in a significant subset of patients (26% of the trial population).^9^ This study compared tivozanib, a potent and selective inhibitor of VEGF receptors 1-3, to sorafenib. It met its primary endpoint of progression-free survival (PFS), achieving a median PFS of 5.6 months with tivozanib versus 3.9 months with sorafenib (hazard ratio [HR] 0.73; *P* =.02) in the intention-to-treat population (ITT). The response rate (RR) was also higher among those in the tivozanib arm (18% vs 8%). Based on these findings, the FDA granted approval to tivozanib for the treatment of relapsed/refractory mRCC following 2 or more prior systemic therapies.^10^ Final overall survival (OS) analyses indicated no clear improvement with tivozanib, showing a nonstatistically significant HR of 0.89 (0.70-1.14; *P* =.35).^11^

Considering the importance of deciding how to treat patients who have previously received CPIs, this analysis brings updated blinded independent central review (BICR) results of OS for the previously stratified population of patients with prior CPI exposure and of duration of response (DOR) in both the ITT population and in those who were CPI-resistant. A preliminary report of these data was previously presented at the ESMO Congress 2024.^12^

## Methods

### Study design

Details of this international trial, which enrolled patients at 120 hospitals across 12 countries, have been previously published.^9^ The study targeted patients with histologically or cytologically confirmed mRCC with a clear cell component presenting measurable disease. Eligibility required having undergone 2 or 3 lines of systemic therapy, including at least 1 VEGF-TKI and an Eastern Cooperative Oncology Group performance status of 0-1.

Patients were stratified and randomized in a 1:1 ratio to receive either tivozanib or sorafenib. Stratification was based on the International mRCC Database Consortium (IMDC) risk score (favorable, intermediate, or poor) and the nature of previous therapies (either two previous VEGF-TKIs, a VEGF-TKI and an immune checkpoint inhibitor (ICI), or a VEGF-TKI and another therapy). The dosing regimen for tivozanib was 1.34 mg orally daily for 3 weeks, followed by a week off (28-day cycle). Sorafenib was administered at 400 mg orally twice daily continuously. Treatment continued until disease progression or the emergence of unacceptable toxicity.

### Assessment of efficacy and toxicity

Patients underwent radiographic imaging every 2 months to monitor efficacy during the treatment phase as specified by the protocol. This imaging was continued until radiographic disease progression was verified by an independent review committee. The effectiveness of the treatment was assessed using the Response Evaluation Criteria in Solid Tumors version 1.1. The study’s primary endpoint was PFS, with OS, RR, and safety as secondary endpoints. Toxicity was evaluated throughout the protocol-based treatment and again 30 days after discontinuing the therapy, using the National Cancer Institute’s Common Terminology Criteria for Adverse Events version 4.03.

### Statistical analysis

In this updated longer follow-up analysis, Cox regression was used to calculate OS results for both the ITT population and patients previously treated with CPIs. Additionally, descriptive statistics were employed to characterize the more recent data on DOR for both tivozanib and sorafenib, again for both the ITT population and those previously treated with CPIs.

## Results

### Patient characteristics

Between May 24, 2016, and August 14, 2017, a total of 350 patients were randomized, with 175 patients allocated to each treatment arm (tivozanib or sorafenib) (Supplementary Figure S1). The patient population was predominantly male (72%), with a median age of 63 (range, 30-90), and primarily white (95%). According to IMDC criteria, the majority had intermediate-risk disease (61%) (Supplementary Table S1).

In terms of prior systemic therapies, a slight majority had received 2 prior therapies compared with 3 (60% vs 40%). Regarding previous CPI treatment, most patients in this group had received a CPI just prior to study inclusion. Approximately a quarter of patients had received both VEGF-TKI and CPI (26%); 47 patients (27%) in the tivozanib group and 44 patients (25%) in the sorafenib arm. Patients from this subset have their characteristics summarized in Table 1. The remainder had received at least 1 VEGF-TKI and other prior systemic agents or 2 prior VEGF-TKIs.

**Table 1.** Patient characteristics.

	Tivozanib *n* = 47	Sorafenib *n* = 44
Median age, years (range)	62 (34–84)	67 (45–87)
Sex—no. (%)		
Female	13 (28)	10 (23)
Male	34 (72)	34 (77)
IMDC risk category—no. (%)		
Favorable	12 (26)	12 (27)
Intermediate	30 (64)	28 (64)
Poor	5 (11)	4 (9)
Histopathology—no. (%)		
Clear cell	44 (94)	38 (86)
Clear cell component	3 (6)	5 (11)
Other	0	1 (2)
Previous treatments—no. (%)		
VEGFR-TKI and VEGFR-TKI	0	0
VEGFR-TKI and immunotherapy	47 (100)	44 (100)
VEGFR TKI and other systemic therapy	0	0
Race—no. (%)		
White	43 (92)	38 (86)
Asian	1 (2)	1 (2)
Black or African American	0	2 (5)
Other/unknown	3 (6)	3 (7)
No. of previous therapies—no. (%)		
2	25 (53)	20 (45)
3	22 (47)	24 (55)

Abbreviations: IMDC, International Metastatic Renal Cell Carcinoma Database Consortium; VEGFR, vascular endothelial growth factor receptor; TKI, tyrosine kinase inhibitor.

### Efficacy results

**Figure 1.**
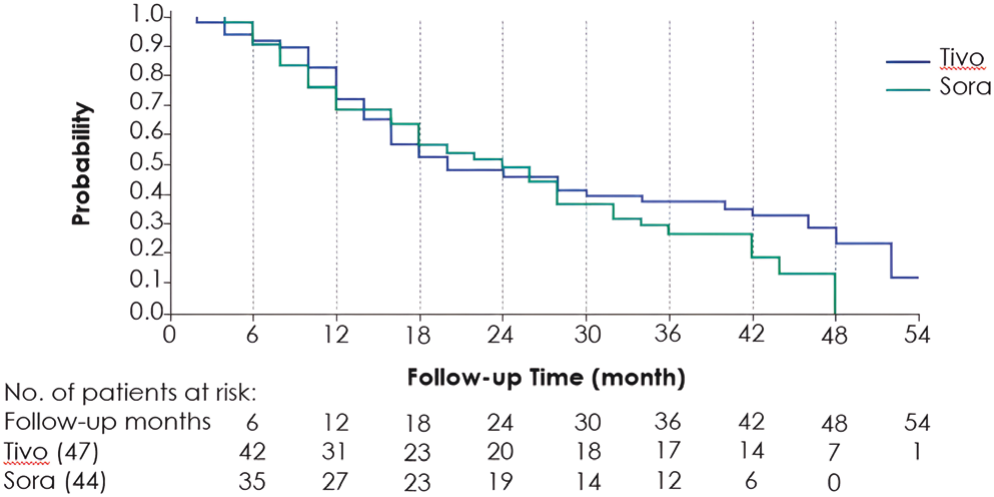
Overall survival in patients receiving prior CPI.

As previously reported for the ITT population, tivozanib significantly improved PFS compared with sorafenib (5.6 vs 3.9 months; *P* =.016), achieving an HR value of 0.73 (95% CI, 0.56-0.94).^9^ In patients who had received prior treatment with both a VEGFR-TKI and a CPI, tivozanib demonstrated a PFS HR of 0.55 (95% CI, 0.32-0.94) and a median PFS of 7.3 months compared with 5.1 months in the sorafenib arm (Table 2). According to the latest BICR assessment with a final cutoff date of June 21, 2021, OS was comparable between the 2 treatment groups in the ITT population, with an HR value of 0.89 (95% CI, 0.70-1.14, *P* =.35) favoring tivozanib. In patients previously treated with CPI, OS also favored the tivozanib arm, with an HR value of 0.69 (95% CI, 0.43-1.11, *P* =.0992) and a median OS of 18.1 and 20.9 months, for tivozanib and sorafenib, respectively (Figure 1).

**Table 2.** Efficacy endpoints by trial arm and prior-CPI exposure.

Patient trial population	Tivozanib		Sorafenib	
	ITT *N* = 175	Prior-CPI *N* = 47	ITT *N* = 175	Prior-CPI *N* = 44
**Median PFS**	5.6 months	7.3 months	3.9 months	5.1 months
**PFS HR (95% CI), *P*-value**	0.73 (0.56, 0.95), *P* =.02	0.55 (0.32, 0.94)		
**Landmark 1-year PFS Rate**	28%	37%	11%	5%
**Median OS**	16.4 months	18.1 months	19.2 months	20.9 months
**OS HR (95% CI), *P*-value**	HR = 0.89 (0.70, 1.14), *P* =.35	HR = 0.69 (0.43, 1.11), *P* =.0992		
**ORR %**	18%	24%	8%	7%
**Median DOR, months**	22.0	20.3	5.7	5.7

Abbreviations: CPI, checkpoint inhibitor; PFS, progression-free survival; HR, hazard ratio; CI, confidence interval; CR, complete response; PR, partial response; SD, stable disease; TTR, time to treatment response; DOR, duration of response.

The RR in the ITT population was 18% for tivozanib, compared with 8% for sorafenib. In patients previously treated with both VEGF-TKI and a CPI, the RR was numerically higher with tivozanib (24% vs 7%). Additionally, the time to treatment response was shorter for tivozanib patients in the CPI-treated subgroup at 2.0 months, versus 3.6 months in the overall ITT population.

Regarding patients who experienced a disease response in the ITT population, the latest data cutoff revealed a median DOR of 22 months (12.7-NR) for tivozanib, compared with 5.7 months (5.6-29.5) for sorafenib. Of those who responded in the tivozanib group, 16 out of 31 (51%) remained progression-free, whereas only 4 out of 14 (28%) did so in the sorafenib group.

For the cohort previously treated with both VEGF-TKI and a CPI, the median DOR for tivozanib was 20.3 months (12.7-NR), in contrast to 5.7 months (5.6-NR) for sorafenib. Of the tivozanib-treated patients who showed a response, 5 out of 11 (45%) from the subgroup of 47 with prior CPI treatment were still free of progression at the last data cutoff. On the other hand, of the 3 sorafenib-treated patients who responded, only 1 remained free of progression among the 44 who had previously received CPI therapy.

### Safety and tolerability

The safety of tivozanib was compared with sorafenib in subgroups stratified by age (<65 [55%], 65-74 [35%], and ≥75 [10%] years) and prior CPI therapy (26% vs 74% without). Patients treated with tivozanib stayed on treatment longer (mean, 11.0 months; range, 0.1-36.9 months) than those treated with sorafenib (mean, 6.3 months; range, 0.2-36.1 months). Treatment duration was consistent across age and prior CPI therapy subgroups. Tivozanib also showed a lower rate of dose modifications than sorafenib across subgroups. In either treatment arm, there were no clinically significant differences in dose reductions or discontinuations based on prior CPI therapy; however, patients with prior CPI therapy had dose holds that were >20% higher compared with those without in both treatment arms (Supplementary Table S2).

Toxicities in the ITT population and in the CPI-treated population are listed in Table 3. In patients with prior CPI-therapy, overall rates of grade ≥3 treatment emergent adverse events (TEAEs) attributed to VEGF-TKI therapy were higher in both arms for patients treated with prior CPI therapy (tivozanib [58%] and sorafenib [67%]) compared with those in the ITT population (tivozanib [46%] and sorafenib [55%]). Hypertension occurred more frequently with tivozanib in patients with prior CPI therapy (35% vs 20 % in the ITT population), whereas rash occurred more frequently with sorafenib in patients with prior CPI exposure (23% vs 8% in the ITT population).

**Table 3.** Grade ≥ 3 TEAE attributed to VEGFR TKI class effects in all patients and patients with prior CPI exposure.

TEAE	Tivozanib		Sorafenib	
	ITT *N* = 173, (%)	Prior-CPI *N* = 47, (%)	ITT *N* = 170, (%)	Prior-CPI *N* = 44, (%)
All TEAE	80 (46)	27 (58)	94 (55)	29 (67)
HTN	35 (20)	16 (35)	23 (14)	5 (12)
Diarrhea	3 (2)	1 (2)	16 (9)	2 (5)
Asthenia	8 (5)	1 (2)	6 (4)	3 (7)
Nausea/Vomiting	0	0	4 (2)	1 (2)
Rash	0	0	13 (8)	10 (23)
PPE	1 (1)	0	17 (10)	5 (12)

Abbreviations: TEAE, Treatment emergent adverse event; ITT, intention to treat; CPI, checkpoint inhibitor; HTN, hypertension; PPE, palmo-plantar dysesthesia.

## Discussion

The phase III TIVO-3 study was the first to demonstrate positive outcomes in the third- and fourth-line treatment of mRCC, including a subset of patients previously treated with CPI. The study showed a statistically significant improvement in PFS with tivozanib compared with sorafenib, an effect that was also seen in patients who had prior exposure to CPI.^9^ Although the median OS was higher in the sorafenib group, extended follow-up of OS has yielded an HR increasingly favoring tivozanib,^11^ albeit not-statistically significant. This shift toward tivozanib is particularly significant in the subset of patients previously treated with CPI, a finding first reported in this analysis.

Extended follow-up data have also shown that patients responding to tivozanib experience a prolonged DOR. Previous long-term PFS data indicated that patients free of progression at the 12-month landmark often derive substantial, durable benefits from tivozanib.^13^ In this previous analysis, with OS data conditioned to patients that achieved at least 12-month of PFS, the HR found was 0.45 (95% CI, 0.22-0.91). Median OS in this subgroup was 48.3 months (95% CI, 32.8-NR) in the tivozanib arm compared with 32.8 months (95% CI, 27.6-50) in the sorafenib arm.^13^

Notably, patients with prior CPI exposure who responded to tivozanib did so more quickly than those who were CPI-naive. At least 2 possible explanations can be attributed to our finding. First, the enhanced efficacy observed in both the tivozanib and sorafenib arms among patients previously exposed to CPIs may stem from residual immunotherapy effects. Second, patients recruited immediately following disease progression after CPI treatment rather than a prior VEGFR-TKI, might exhibit heightened responsiveness to any VEGFR-TKI. This heightened response could potentially be attributed to the restoration of reversible resistance mechanisms to VEGFR inhibition, particularly within the tumor microenvironment. Alternatively, resistance might have developed due to the formation of tumor vasculature that is less dependent on VEGF-signaling, which could eventually reemerge after treatment breaks.^14-17^

These findings underscore the effectiveness of tivozanib as a treatment option in the CPI-resistant setting. In parallel, belzutifan has already emerged as an alternative for a similar patient group.^8^ However, any cross-trial comparisons would be extremely challenging, as the LITESPARK-005 and TIVO-3 studies differ in several respects, including extent of prior therapy and choice of control arm.^18^ From this standpoint, information on how to identify which patients might benefit most from each treatment and how to effectively sequence them is an issue to be debated.

An unresolved issue in the CPI-resistant setting is the potential for re-exposure to immunotherapy. Initially, promising data came from a phase II single-arm trial of pembrolizumab and lenvatinib, which demonstrated high objective RRs.^19^ However, enthusiasm for this strategy waned following the negative results of the CONTACT-03 trial. This trial compared the combination of atezolizumab with cabozantinib to cabozantinib alone, showing superimposable PFS curves.^20^ The TiNivo-2 study employed a similar overarching design, comparing tivozanib with or without nivolumab in patients receiving prior CPI.^21^ The study does have several methodologic differences. As one example, TiNivo-2 allows for patients who have intervening treatments between CPI and tivozanib-based therapy and furthermore uses different doses on the control arm (1.34 mg daily, 3 weeks on, 1 week off) versus the experimental arm (0.89 mg daily, 3 weeks on, 1 week off with nivolumab monthly). While the study reportedly failed to meet its primary endpoint, demonstrating improved PFS with the combination, it ultimately provided further prospective data related to tivozanib in a CPI-treated population.^22^

In terms of safety and tolerability, the trial indicates that tivozanib has a more favorable side effect profile compared with sorafenib. While the total incidence of grade ≥3 TEAEs was similar between the 2 treatment arms, tivozanib was associated with a longer treatment duration and fewer dose reductions, interruptions, and discontinuations.^23^ This trend was observed even in patients with prior CPI exposure, although toxicities were more prevalent in this patient population in both treatment arms. Of special interest, the most common adverse event in the tivozanib arm was hypertension, which was proportionately higher in the subgroup with prior CPI exposure. In the TiNivo phase 1b study, which tested 25 patients with the combination of tivozanib and nivolumab, there was an unexpectedly high rate of grade ≥3 hypertension, occurring in 52% of patients.^24^ As such, lingering effects from immunotherapy could also justify the increased rates of hypertension found in the present population treated with tivozanib who were previously exposed to CPI. This possible association was not evident with the results from TiNivo-2, although the different doses in the 2 arms of the trial may have attenuated this effect.^22^

The study’s limitations include the small sample size of the subset of patients previously treated with CPIs and its post-hoc status, which entails the absence of formal statistical validation. Therefore, the generalizability of these results should be approached with caution. Nonetheless, it does seem that tivozanib maintains its efficacy in heavily pretreated patients and, due to its favorable toxicity profile, it strengthens its position as a treatment option even after CPI failure.

In conclusion, the current analysis indicates that, with longer follow-up, tivozanib trended toward improved OS compared with sorafenib, especially in the subset of patients previously treated with CPIs.

## Supplementary Material

oyae369_suppl_Supplementary_Figures_1

oyae369_suppl_Supplementary_Tables_1-2

## Data Availability

The data underlying this article were provided by Aveo Oncology. Data will be shared on request to the corresponding author with permission of Aveo Oncology.
