# Supplementary figures and images for: Updated overall survival in patients with prior checkpoint inhibitor therapy in the phase III TIVO-3 study
Source: Oncologist. 2025 Feb 6;30(2):oyae369. doi: 10.1093/oncolo/oyae369 (PMC11799859; doi:10.1093/oncolo/oyae369)

**SUPPLEMENTARY DATA**

**Supplementary Figure 1: Consort diagram**


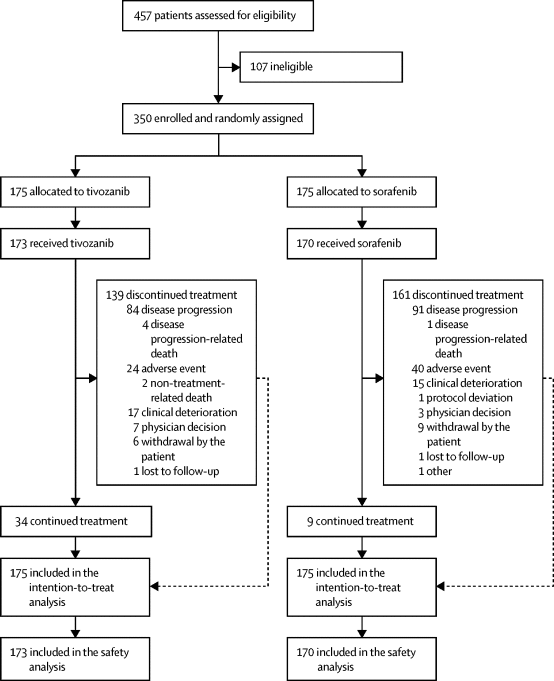

Supplement: oyae369_suppl_Supplementary_Figures_1 [file oyae369_suppl_supplementary_figures_1.docx]
