# Supplementary material for: Updated overall survival in patients with prior checkpoint inhibitor therapy in the phase III TIVO-3 study
Source: Oncologist. 2025 Feb 6;30(2):oyae369. doi: 10.1093/oncolo/oyae369 (PMC11799859; doi:10.1093/oncolo/oyae369)
Supplement: oyae369_suppl_Supplementary_Tables_1-2 [file oyae369_suppl_supplementary_tables_1-2.docx]

**Supplementary Table 1.** Patient characteristics.

| **Patient characteristics** | **Tivozanib**  **n=175** | **Sorafenib**  **n=175** |
| --- | --- | --- |
| Median age, years (range) | 62 (34-88) | 63 (30-90) |
| Sex – no. (%) |  |  |
| Female | 49 (28) | 47 (27) |
| Male | 126 (72) | 128 (73) |
| IMDC risk category – no. (%) |  |  |
| Favorable | 34 (19) | 36 (12) |
| Intermediate | 109 (62) | 105 (60) |
| Poor | 32 (18) | 34 (19) |
| Histopathology – no. (%) |  |  |
| Clear cell | 165 (94) | 160 (91) |
| Clear cell component | 9 (5) | 9 (5) |
| Other | 1 (1) | 5 (3) |
| Previous treatments – no. (%) |  |  |
| VEGFR-TKI and VEGFR-TKI | 79 (45) | 80 (46) |
| VEGFR-TKI and immunotherapy | 47 (27) | 44 (25) |
| VEGFR TKI and other systemic therapy | 49 (28) | 51 (29) |
| Race – no. (%) |  |  |
| White | 165 (94) | 167 (95) |
| Asian | 2 (1) | 2 (1) |
| Black or African American | 0 | 2 (1) |
| Other/unknown | 8 (5) | 4 (2) |
| No of previous therapies – no. (%) |  |  |
| 2 | 108 (62) | 104 (59) |
| 3 | 67 (38) | 71 (41) |
| IMDC, International Metastatic Renal Cell Carcinoma Database Consortium; VEGFR, vascular endothelial growth factor receptor; ITT, intention to treat; TKI, tyrosine kinase inhibitor. | | |

**Supplementary table 2.** Dose modifications by age and prior immuno-oncology therapy status

|  | | **Drug exposure, mean cycles, n** | **Dose interruption, %** | **Dose reduction, %** | **Dose discontinuation, %** |
| --- | --- | --- | --- | --- | --- |
| **All** | Tivozanib (n=173) | 11.9 | 48 | 24 | 21 |
|  | Sorafenib (n=170) | 6.7 | 64 | 39 | 30 |
| **Age <65 y** | Tivozanib (n=97) | 10.1 | 41 | 20 | 26 |
|  | Sorafenib (n=92) | 6.7 | 51 | 26 | 25 |
| **Age 65-74 y** | Tivozanib (n=61) | 15.0 | 59 | 33 | 15 |
|  | Sorafenib (n=59) | 6.6 | 80 | 51 | 36 |
| **Age ≥75 y** | Tivozanib (n=15) | 11.2 | 73 | 33 | 20 |
|  | Sorafenib (n=19) | 7.3 | 74 | 63 | 37 |
| **Prior CPI** | Tivozanib (n=47) | 12.3 | 69 | 33 | 25 |
|  | Sorafenib (n=44) | 5.5 | 81 | 35 | 40 |
| **No. of prior CPI** | Tivozanib (n=126) | 11.7 | 43 | 22 | 20 |
|  | Sorafenib (n=126) | 7.2 | 57 | 40 | 27 |
